# Correction: From micro/nano structured isotactic polypropylene to a multifunctional low-density nanoporous medium

**DOI:** 10.1039/d3ra90055j

**Published:** 2023-06-26

**Authors:** Mehdi Saniei, Minh-Phuong Tran, Seong-Soo Bae, Piyapong Buahom, Pengjian Gong, Chul B. Park

**Affiliations:** a Microcellular Plastics Manufacturing Laboratory, Department of Mechanical and Industrial Engineering, University of Toronto 5 King's College Road Toronto Ontario M5S 3G8 Canada park@mie.utoronto.ca

## Abstract

Correction for ‘From micro/nano structured isotactic polypropylene to a multifunctional low-density nanoporous medium’ by Mehdi Saniei *et al.*, *RSC Adv.*, 2016, **6**, 108056–108066, https://doi.org/10.1039/C6RA22607H.

The authors regret that the name of one of the authors (Piyapong Buahom) was shown incorrectly in the original article. The corrected author list is as shown above.

The Royal Society of Chemistry apologises for these errors and any consequent inconvenience to authors and readers.

## Supplementary Material

